# The British Soldier Turned Teetotaler

**Published:** 1888-10-20

**Authors:** 


### THE BRITISH SOLDIER TURNED TEETOTALER.

The British soldier has at last discovered that he can fight on water. It is stated that in India the regimental canteen is disappearing before the regimental coffee-shop. Last year, out of 1,146 courts-martial held in Bengal 392 were distinctly traceable to drunkenness. In the Madras Presidency more than a fourth of all the courts-martial were due to the same cause. The percentage of cases wherein temperance men were contaminated is said to have been insignificant. Official statistics also prove the comparative immunity of temperance men from sickness and disease. The supreme Government has sanctioned the establishment of an institute for every British regiment and battery. The institute will consist of a series of well-lighted rooms, and a part of it will be devoted to a coffee-shop, from which total abstainers and temperance men will be able to have a comfortable meal in a separate room. In every sense the soldier is improved by the change. "Thrift, good order, and efficiency" are the universal accompaniments of temperance.

